# Nanoscale temperature mapping through thermal vibration characterization using scanning precession electron diffraction

**DOI:** 10.1126/sciadv.aeb9234

**Published:** 2026-02-13

**Authors:** Kun Yang, Chao Zhang, Chengwei Wu, Qian Du, Bingzhi Li, Zhen Fang, Liang Li, Peng Wang, Wen Shang, Jianbo Wu, Tianru Wu, Hui Wang, Tao Deng, Wenpei Gao

**Affiliations:** ^1^ State Key Laboratory of Metal Matrix Composites, School of Materials Science and Engineering, Future Material Innovation Center, Zhangjiang Institute for Advanced Study, Shanghai Jiao Tong University, Shanghai 200240, China.; ^2^ School of Physics and Electronics, Hunan Key Laboratory of Super Microstructure and Ultrafast Process, State Key Laboratory of Powder Metallurgy, Central South University, Changsha 410083, China.; ^3^ Research Center for Scientific Data Hub, Zhejiang Lab, Hangzhou 310000, China.; ^4^ State Key Laboratory of Dark Matter Physics, Key Laboratory for Particle Astrophysics and Cosmology, Shanghai Key Laboratory for Particle Physics and Cosmology, School of Physics and Astronomy, Shanghai Jiao Tong University, Shanghai 200240, China.; ^5^ Department of Physics, University of Warwick, Coventry, UK.; ^6^ School of Mechanical Engineering, Shanghai Jiao Tong University, Shanghai 200240, China.; ^7^ Institute of Information Functional Materials, Shanghai Key Laboratory of Atomic-Level Intelligent Manufacturing of Materials and Devices, Shanghai Jiao Tong University, Shanghai 200240, China.

## Abstract

Accurate temperature measurement with a high spatial resolution is essential for understanding thermal behavior in integrated nanoscale devices and especially at heterogeneous interfaces. However, existing techniques are often limited by insufficient spatial resolution. Here, we showcase the direct and noncontact temperature measurement with a nanometer spatial resolution using transmission electron microscopy. The experimental probe is the combination of a scanning nanobeam with precession electron diffraction, which offers the collection of kinematic diffraction intensity from a local area at the nanometer scale. With a precalculated, sample- and geometry-specific structure factor–based correction, the linear fitting of diffraction intensities allows the determination of the Debye-Waller factor and, thus, temperature with a precision of 10^−4^ square angstrom per °C. Using graphene as a model material, this work reveals the influence of sample tilt, lattice thermal expansion, and sample thickness on Debye-Waller factor and offers a route to improving the measurement precision along with spatial resolution. The approach establishes a broadly applicable strategy for nanoscale thermometry in low-dimensional and heterogeneous materials.

## Teaser

Direct and noncontact temperature measurement with a nanometer spatial resolution is realized using structure factor correction.

## INTRODUCTION

Thermal management in microelectronics has become a critical bottleneck as semiconductor devices continue to shrink in size and grow in complexity (*1*, *2*). Mapping local temperature at the nanoscale is essential for evaluating the device performance, especially at thermal interfaces (*3*, *4*). However, accurately measuring local temperature and mapping its distribution remain a challenge. Temperature measurement often involves indirect quantification through related physical quantities. Traditionally, thermometric methods can be categorized into contact and noncontact types (*5*). Thermocouples fabricated into fine tips and integrated to atomic force microscopy can probe the temperature of a sample surface (*6*) but require physical contact and time for thermal equilibrium and can introduce thermal disturbance. The spatial resolution is also limited by the physical size of the probe. Noncontact optical thermography techniques, including Raman spectroscopy (*7*), infrared spectroscopy (*8*), and fluorescence (*9*), do not require physical contact but are constrained by the diffraction limit (*10*), resulting in their spatial resolution of about hundreds of nanometers to micrometers, larger than the size of state-of-the-art semiconductor functional units (*11*).

Overcoming these resolution limits requires techniques capable of probing temperature at the nanoscale. Transmission electron microscopy (TEM) offers such capability with a high spatial resolution in materials characterization and may provide a viable pathway toward high-resolution, nanoscale temperature mapping. Available works have shown that TEM can probe materials temperature by quantifying the nanoscale volumetric change of liquid metal confined in carbon nanotubes (*12*) or by characterizing the lattice thermal expansion of metallic nanoparticles (*13*). On the basis of atomic resolution images, Zhu and Hwang (*14*, *15*) reported the possible quantification of local temperature in oxide by the attenuation or enhancement of atomic column intensity in high-angle annular dark-field images. In addition, in TEM, temperature can also be quantified using localized plasmon or phonon excitation in electron energy loss spectroscopy (*16*–*20*). However, most reported approaches can only be applied to samples of a specific type because of the detection or quantification limit of spectroscopy and imaging methods. These methods are either indirect or still limited in resolution. A direct thermometric method with a high spatial resolution is still missing in nanoscale metrology. Compared to the imaging and spectroscopic methods, electron diffraction is simple to perform and less likely to be limited by the sample selection. The only disadvantage is that electron diffractions typically do not offer a high spatial resolution because of the size of the electron beam. With recent advances in aberration correction, an electron probe with the size as small as below 1 Å can be formed by converging the electron beam, offering a routine imaging resolution down to the deep atomic scale. Physical properties that can be probed with electron scattering should also be mappable at such a resolution (*21*). It is worth noting that the Debye-Waller factor is directly related to temperature and can be measured by the diffraction method (*22*–*24*). When combined with a nanometer-sized electron probe, it is possible to probe the sample temperature at both a high spatial resolution and improved precision.

In this work, we report the measurement and mapping of local temperature using four-dimensional scanning TEM (4D-STEM) with a precessing beam. This method collects precession electron diffraction (PED) patterns during probe scanning. By applying sample specific correction factors that consider the structure, tilt, and thickness, a linear fit using the intensity computes the Debye-Waller factor directly, enabling temperature mapping and the probing of local vibration characteristics. Results from graphene show the capability of this method that can measure the Debye-Waller factor and, therefore, temperature at a near–1-nm spatial resolution. Using lattice parameters and thickness determined in the same experiment, and combined with theoretical calculations, the work also reveals detailed insight into the vibrational properties governed by temperature, lattice, and graphene layer thickness.

## RESULTS

### Corrected Wilson plot for PED-based Debye-Waller factor measurement

**Fig. 1.**
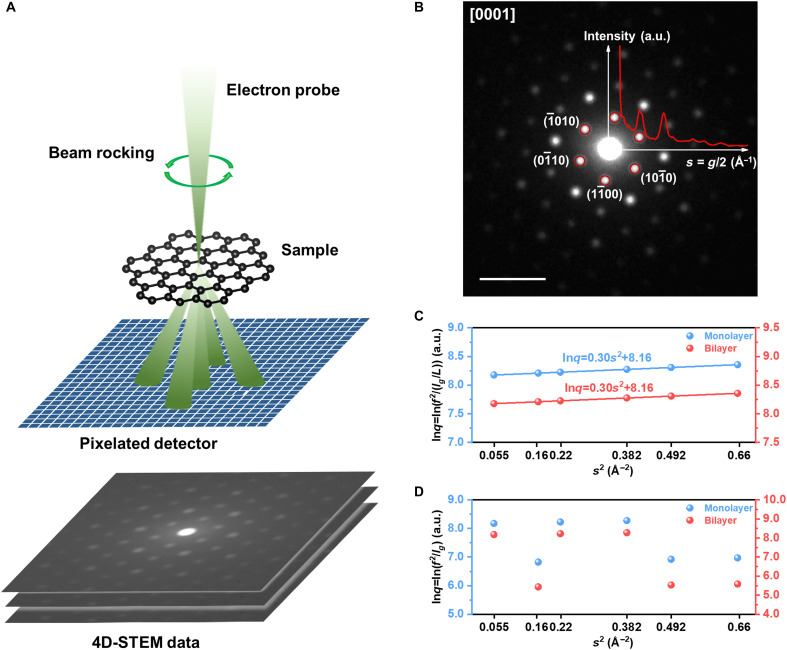
Schematic illustration of scanning PED and the corrected Wilson plot for Debye-Waller factor calculation. (**A**) Experimental setup for 4D scanning electron diffraction using aberration-corrected STEM. (**B**) Average diffraction pattern from the 4D electron diffraction dataset and intensity profile of the pattern. Scale bar, 10 1/nm. a.u., arbitrary units. (**C**) Wilson plot of monolayer graphene and AB-stacking bilayer graphene using the correction factor *L* from Table 1 and results of linear fitting. (**D**) Wilson plot without the correction factor *L*.

Figure 1A shows the experimental setup of scanning PED by combining 4D-STEM with PED. Unlike conventional electron diffraction or imaging, during the raster scanning of the electron probe, the electron beam precesses about the optical axis at a constant angle at each probe position, forming a PED pattern in the back focal plane. Each PED pattern is collected using a hybrid pixelated electron detector operated at the acquisition rate faster than 1000 frames per second. Figure 1B shows the PED pattern of a freestanding monolayer graphene. In electron diffraction analysis, a major obstacle is the dynamical effects that originate from electrons being scattered multiple times by the sample. Intensities in the dynamic diffraction patterns do not simply follow the kinematical theory, making property analysis based on the distribution of diffraction intensity challenging. Here, the net effect of PED is equivalent to the sample being precessed relative to a stationary axis. By averaging many reflections that satisfy Bragg’s law (*25*), ultimately, the effect of dynamic diffraction can be reduced and the diffraction intensity follows that of the kinematic diffraction. In kinematical theory, the *g*_th_ order diffraction intensity, $I_{g}$, is proportional to the magnitude of the structure factor, F(hkl), expressed as $I_{g}\propto \langle {|F\left(\mathrm{hkl}\right)|}^{2}\rangle$. The structure factor also includes the Debye-Waller factor, commonly written as $e^{-Bs^{2}}$, which accounts for the attenuation of diffraction intensity resulting from thermal vibrations of atoms. Here, *B* is often referred to simply as the Debye-Waller factor. We can express the diffraction intensity $I_{g}$ as

$$KI_{g}=e^{-2Bs^{2}}f^{2}\left(s\right){\left[\sum_{j=1}^{n}e^{2\pi i\left(hx_{j}+ky_{j}+lz_{j}\right)}\right]}^{2}$$ (1)

where K is a scaling constant, f(s) is the atomic scattering factor, and $\left(x_{j},y_{j},z_{j}\right)$ are the fractional coordinates of the *j*th atom in the unit cell. Detailed derivations and definitions can be found in (*26*–*30*) and are also provided in the Supplementary Materials with discussions.

Compared to other methods in temperature measurement (*14*, *15*), the Debye-Waller factor reflects the temperature-dependent phonon population, making it directly related to temperature. Therefore, measuring the Debye-Waller factor provides a direct means of determining the sample’s temperature. While the Debye-Waller factor can usually be measured using x-ray and neutron diffraction, these methods require appropriate absorption and extinction correction and rigorous data fitting (*31*–*33*). Both x-ray and neutron scattering techniques are also limited by a spatial resolution of hundreds of nanometers to micrometers at best. On the other hand, an accurate ab initio calculation of the Debye-Waller factor requires an accurate value of the elastic constant and quantification of anharmonic effects, both of which are difficult to obtain (*34*–*37*).

To directly estimate the Debye-Waller factor from diffraction, Wilson (*22*), Yü (*23*), and Bragg (*24*) proposed that if we assume a random distribution of atoms in a unit cell, the log of intensity, $I_{g}$, divided by the summation of atomic scattering factors, $f^{2}\left(s\right)$, is proportional to the square of the scattering vector, $s^{2}$, i.e., $\text{ln}q=2Bs^{2}+\text{ln}K,$ where $q=f^{2}\left(s\right)/\left(I_{g}/L\right)$, K is a scaling factor, and $L\;=n^{2}$, where n is the number of atoms in the unit cell. On the basis of this, in the Wilson plot that reflects the x-ray diffraction intensity as a function of the scattering vector, lnq and $s^{2}$ can be plotted as a straight line with a slope of 2*B*. Midgley *et al.* (*38*) extended this method in electron diffraction to measure the Debye-Waller factor from higher-order Laue zone reflections obtained by PED.

Using this method, however, even for crystals of a single element, the Wilson plot does not strictly satisfy a linear fit, because the assumption of random distribution of atoms in the unit cell ignores the phase information in the diffraction caused by the positions of the atoms, resulting in the error in the value of *L*, leading to systematic errors that cannot be corrected without additional information. Here, we show that because the *L* from the structure factor can be precisely calculated, the effect of *L* can be compensated using a correction factor 1/*L* that is determined only by the reciprocal lattice index, the shape of the relrod, and the sample thickness. In this work, we use monolayer graphene and AB-stacking bilayer graphene as model materials and measure the local temperature using the Debye-Waller factor calculated from the corrected Wilson plot.

We first calculate the correct *L*, as $L={\left[\sum_{j=1}^{n}e^{2\pi i\left(hx_{j}+ky_{j}+lz_{j}\right)}\right]}^{2}$. The value for monolayer and AB-stacking bilayer graphene is listed in Table 1. By plotting the value, $\text{ln}q=\text{ln}\left[f^{2}\left(s\right)/\left(I_{g}/L\right)\right]$, with the correct *L*, against $s^{2}$, the Wilson plot is linear in Fig. 1C. For comparison, Fig. 1D shows the Wilson plot without such a correction, which does not afford for high-quality linear fit. On the basis of the slope of the straight line in Fig. 1C, we can directly obtain the Debye-Waller factor from a diffraction pattern.

**Table 1.** *L* of *g*_th_ diffraction for monolayer graphene and AB-stacking bilayer graphene.

Crystal	1st	2nd	3rd	4th	5th	6th
Monolayer	1	4	1	1	4	4
AB bilayer	1	16	1	1	16	16

### Experimental setup and acquisition optimization for PED measurements

**Fig. 2.**
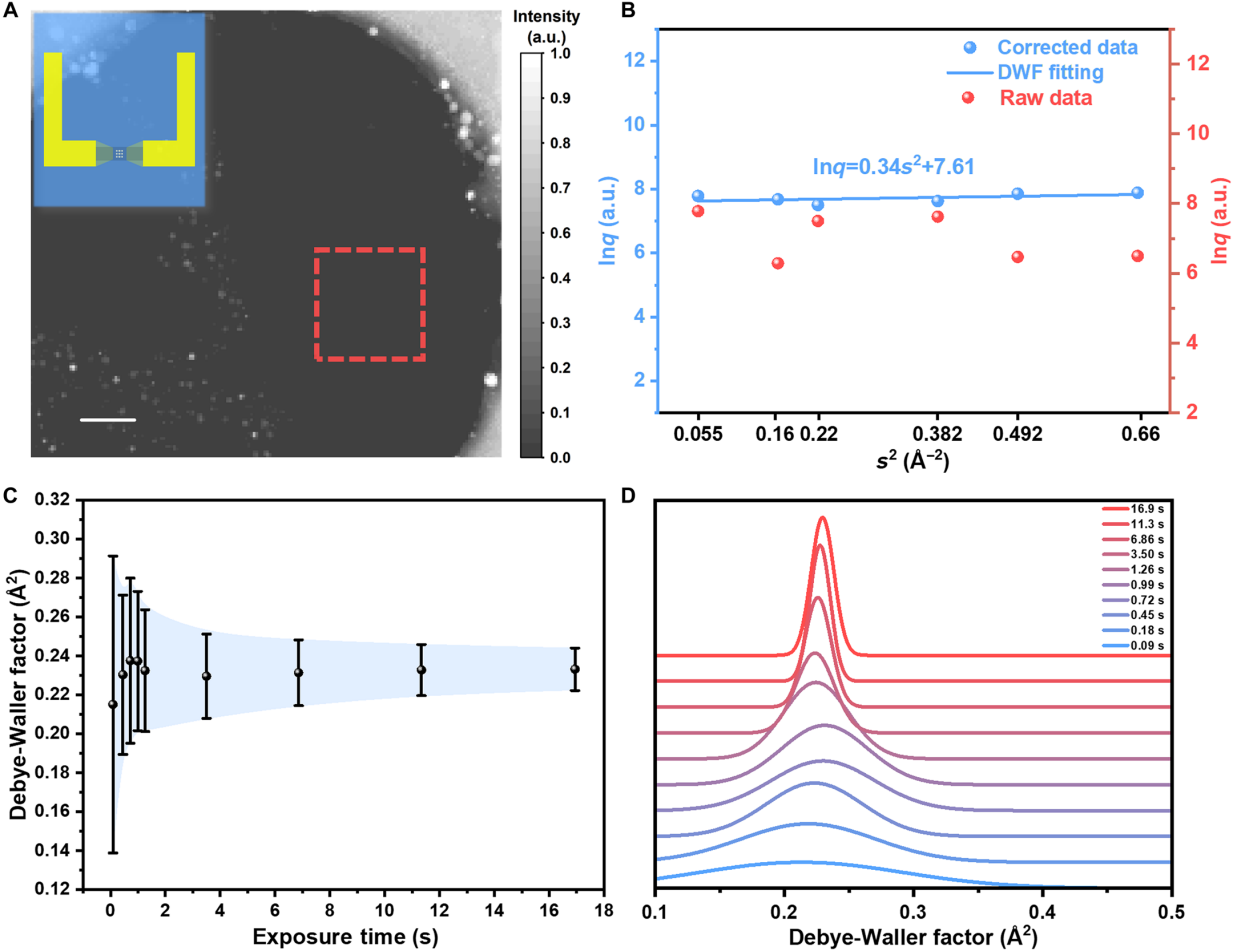
Influence of frame acquisition time on Debye-Waller factor measurement. (**A**) Annular dark-field image from 4D electron diffraction data by integrating the intensity of the annular region of 10 to 75 mrad from the diffraction pattern in Fig. 1B. Scale bar, 60 nm. (**B**) Wilson plot of the average diffraction intensities. DWF, Debye-Waller factor. (**C**) Measured Debye-Waller factor as a function of exposure time. (**D**) Fitted Gaussian of Debye-Waller factors measured with different acquisition times.

In experiment, monolayer and bilayer graphene samples are grown using a customized chemical vapor deposition (CVD) method (*39*). The graphene sample was transferred to a microelectromechanical system–based heating chip from Protochips Inc. for scanning PED from room temperature to 950°C. Using Thermo Fisher Scientific Spectra 300 aberration-corrected STEM operated at 300 kV, the scanning PED was carried out by taking 4D-STEM using TopSpin from NanoMEGAS under the microprobe diffraction mode with an aperture of 50 μm in diameter and different precession angles (0.02° for monolayer and bilayer graphene and 2° for thicker graphite). The size of the scanning probe is measured to be about 1.4 nm. The convergence angle used in PED experiment is about 0.89 mrad. Each PED pattern is acquired with a Merlin hybrid pixel camera at the rate of 1000 frames per second. In a scanning PED experiment, we acquire diffraction patterns in 170-by-170 scanning positions with a step size of 2 to 3 nm, corresponding to a region of 340 nm by 340 nm or 510 nm by 510 nm in size. The reconstructed annular dark-field image obtained from a graphene area is shown in Fig. 2A. A region with clean, uniform image contrast is selected as indicated by the red dashed box in Fig. 2A, and the average diffraction pattern is shown in Fig. 1B. The average diffraction intensities are plotted as a Wilson plot in Fig. 2B; similar to Fig. 1 (C and D), the corrected Wilson plot (in blue) fits much better with a linear relationship than the uncorrected one (in red). In each PED, the center position of the diffraction spots is first determined using circle edge fitting in AutoDisk (*29*) and the center-of-mass method. For each diffraction spot, the radius is determined to be 4 pixels, within which the total intensity is counted as the diffraction intensity; details can be found in fig. S1. Using the *L* listed in Table 1 for monolayer graphene and with additional correction on the basis of surface topology, a linear fit of the Wilson plot calculates the Debye-Waller factor from each diffraction pattern acquired at each location.

To determine the acquisition time for each frame of diffraction pattern, we collected data with different frame exposure times in the same area at a constant temperature (800°C). As shown in Fig. 2 (C and D) and fig. S2, from 0.09 to 1.26 s, a longer exposure time results in lower standard deviation of the measured Debye-Waller factor, from 0.076 to 0.031 Å^2^, because of the improvement of signal-to-noise ratio. By averaging the data through 3-by-3 to 11-by-11 binning equivalent to a 16.9-s exposure time, the standard deviation drops to 0.011 Å^2^. A further increase in exposure time or frame binning does not show much improvement in standard deviation, and the distribution of the Debye-Waller factor may then reflect the temperature fluctuation across the sample.

### Local tilt mapping for accurate Debye-Waller factor measurement

Note that freestanding graphene (*40*–*42*) may not be perfectly flat, and ripples and surface undulations are commonly observed. These humps and depressions introduce local curvature or tilt to the surface. Although the Bragg peaks of ideal two-dimensional materials extend into nearly infinite reciprocal-space rods perpendicular to the specimen surface, real structural deviations, such as curvature, tilt, and variations in atomic stacking, modify both the intensity and phase of each relrod, thereby altering the measured diffraction intensity (*43*). As a result, the apparent fluctuations in the Debye-Waller factor measured at a constant temperature may reflect not only real local temperature variations but also surface morphology effects. To accurately determine the Debye-Waller factor from diffraction intensities, both the intrinsic shape of the relrod and its intersection with the Ewald sphere must be considered.

**Fig. 3.**
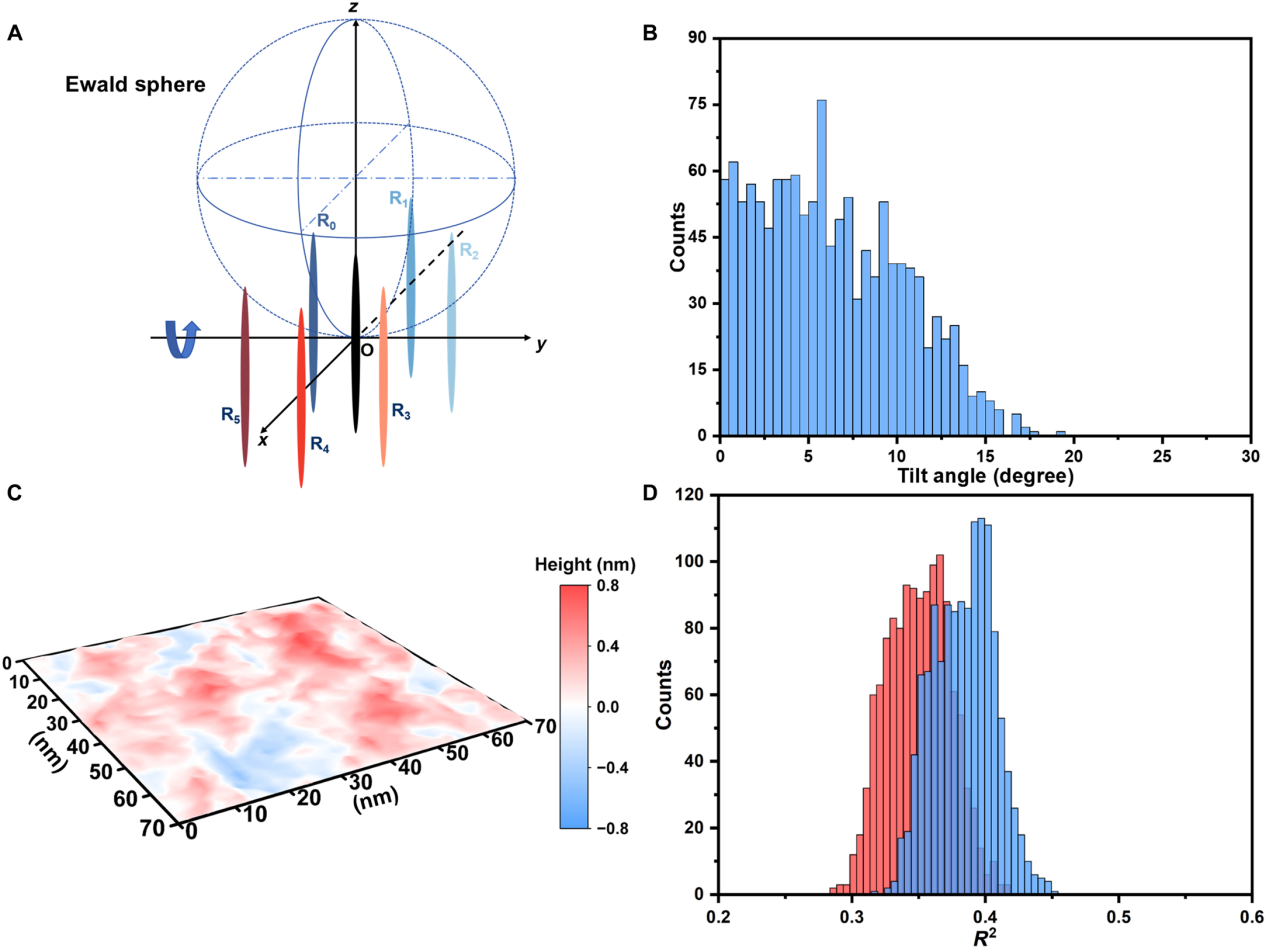
Influence of surface topography of monolayer graphene on Debye-Waller factor measurement. (**A**) Schematic of the Ewald sphere intersecting the Bragg rods. (**B**) Histogram of tilt angle from the select area and at 800°C. (**C**) Surface topology of monolayer graphene at 800°C. (**D**) *R*^2^ value of the Debye-Waller factor before (red) and after (blue) correction using the shape factor.

Quantifying the influence of surface morphology requires estimating the local tilt angle of monolayer graphene. We start from analyzing the asymmetry in diffraction disk intensities. As illustrated in Fig. 3A, the Ewald sphere intersects the reciprocal rods, which are elongated because of the finite thickness and two-dimensional nature of graphene (*44*). This elongation causes the intensity of each diffraction spot to depend on its distance from the Ewald sphere, a distance that changes systematically with local lattice tilt. Additional schematic details are provided in fig. S3. We establish a quantitative reference by calculating the three-dimensional Fourier transform of monolayer graphene using atomic coordinates and electron densities obtained from density functional theory. While the reciprocal rods extend over a long range in the reciprocal space, the Bragg peak intensities attenuate with increasing scattering vector because of the atomic form factor. For a series of known tilt angles, we simulated diffraction patterns and calculated the intensity distribution of same-order diffraction spots located on different sides of the tilt axis. This produced a calibration curve relating the intensity ratio to the tilt angle (fig. S3A).

For the experimental 4D-STEM data, each diffraction pattern was divided into six angular sectors (fig. S3B), reflecting the hexagonal symmetry of graphene. The tilt axis was identified as the direction along which the diffraction pattern exhibited approximate mirror symmetry. For all three pairs of opposing diffraction spots straddling this axis, we computed the intensity ratio. By matching these experimental ratios to the simulated calibration curve, we extracted the local tilt angle and generated a spatial tilt map for the area shown in Fig. 2A. A histogram of the tilt angle distribution is shown in Fig. 3B. The freestanding monolayer graphene imaged hosts ripples with inclined surfaces at angles as high as 15°, with most populated from 0° to 10°. Combining the tilt angles with their corresponding axes allowed us to reconstruct the surface ripple morphology of monolayer graphene, as shown in Fig. 3C.

Local curvature and ripples modify the geometric conditions for diffraction, causing the correction factors in Table 1 to deviate from their nominal values and introducing systematic errors into Debye-Waller factor measurements. To account for this effect, we applied an additional correction to the values in Table 1 using the calibration curve (fig. S3) together with the measured tilt angles. After this correction, the *R*^2^ value of the linear fit for Debye-Waller factor determination improved substantially (Fig. 3D), confirming that compensating for local tilt produces a more accurate linear relationship. Furthermore, when the local surface is tilted, out-of-plane thermal vibrations (along the *z* axis) are projected into the *x*-*y* plane so that the measured Debye-Waller factor contains contributions from both in-plane and out-of-plane atomic motions.

### Temperature mapping of graphene using scanning PED

**Fig. 4.**
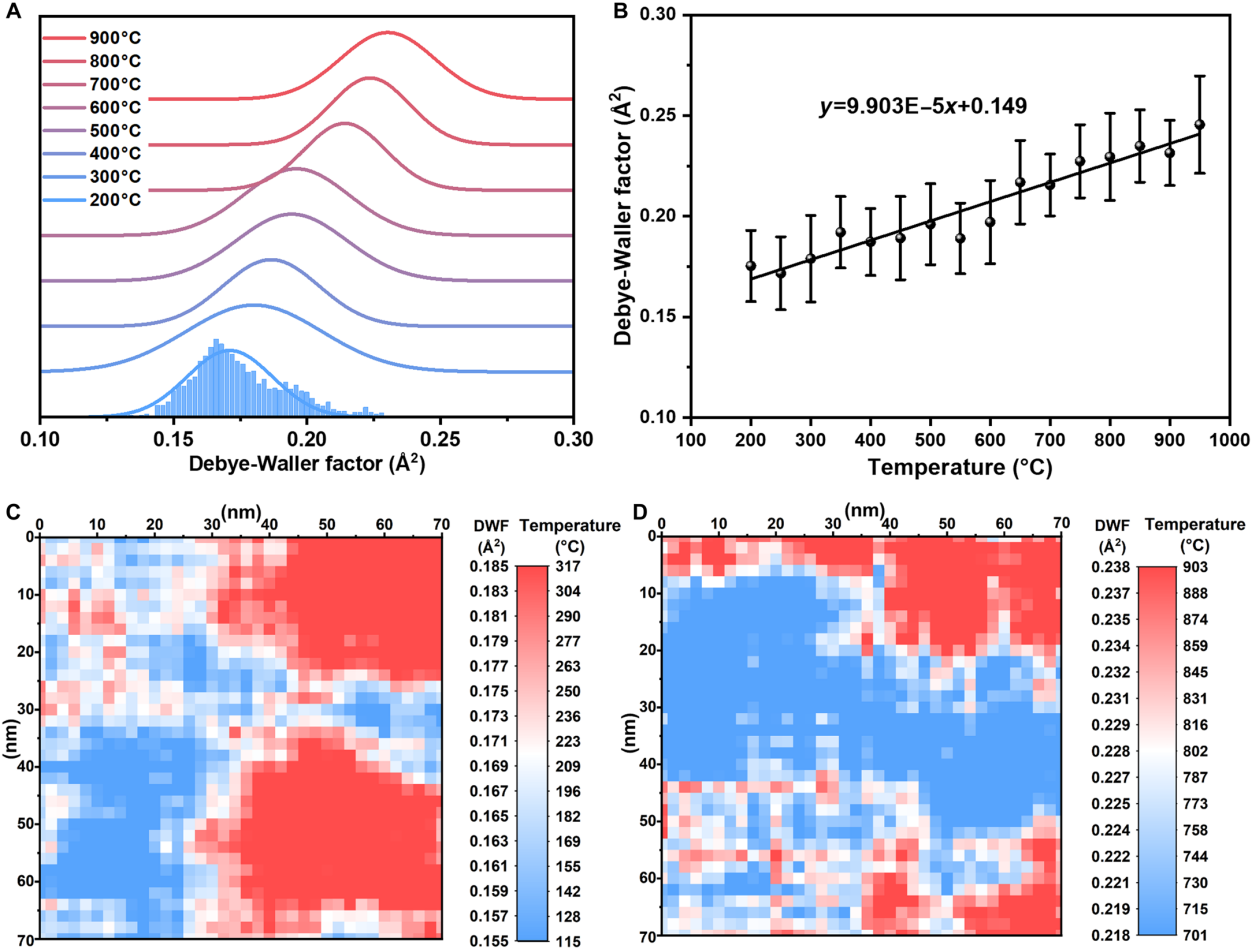
Scheme illustration of the measurement of the Debye-Waller factor at different temperatures. (**A**) Gaussian-fitting curve of different temperatures and histogram of the red dashed area in Fig. 2A at a specified temperature (200°C). (**B**) Debye-Waller factor of monolayer graphene as a function of temperature, ranging from 200° to 950°C. (**C**) Mapping of the Debye-Waller factor and temperature of the red dashed area in Fig. 2A at 200°C. (**D**) Mapping of the Debye-Waller factor and temperature of the red dashed area in Fig. 2A at 800°C.

With the calibration curve, we can map the temperature distribution in a scanning PED experiment. At each specified temperature (e.g., 200°C), the Debye-Waller factors obtained from 1600 diffraction patterns in the red dashed area were counted and plotted as a histogram, as shown in Fig. 4A, and fitted using Gaussian. The centers of Gaussian-fitted Debye-Waller factor distribution shift toward a higher value with temperature.

The Debye-Waller factors, when plotted against temperature, show a clear linear relationship, with a slope of 9.903 × 10^−5^ Å^2^/°C, as shown in Fig. 4B. The histogram of the Debye-Waller factor from 200° to 900°C is shown in fig. S4. At 200° and 800°C, the mapping of the Debye-Waller factor and temperature within the red dashed box in Fig. 2A is shown in Fig. 4 (C and D). In the experimental setup, the scanning PED offers a spatial resolution of about 1.4 nm, so temperature measurements can be obtained at the same resolution. It is seen that despite the temperature set for the microelectromechanical system heating chip, the population and distribution of temperature in the mapped area are rather scattered. Although, in both the map and histogram at 800°C (Fig. 4D), most Debye-Waller factors concentrate around 0.229 Å^2^, they can range from 0.218 to 0.238 Å^2^, corresponding to the temperature from 701° to 903°C. The fluctuation of the Debye-Waller factor value indicates that temperature is not uniform across the mapped area in graphene but is higher on the top and bottom areas and lower in the center. Depending on whether the correction factors considering the influence of the tilt angle are used, the measured Debye-Waller factor value varies slightly as shown in fig. S5.

We also estimated the effect of electron beam heating, which can be expressed as $∆T\approx I_{0}/e\kappa \cdot \mathrm{dE}/\mathrm{dx}$ (*45*–*47*), where $I_{0}$ is the beam current (15 pA), *dE*/*dx* is the energy loss rate per electron (0.15 eV/nm), and κ is the thermal conductivity [5000 W m^−1^ K^−1^ for graphene (*48*)]. This value, 4.5 × 10^−7^ K, is much smaller than the temperature measured on the basis of diffraction intensity and hence can be ignored.

### Influence of thermal expansion on the Debye-Waller factor

**Fig. 5.**
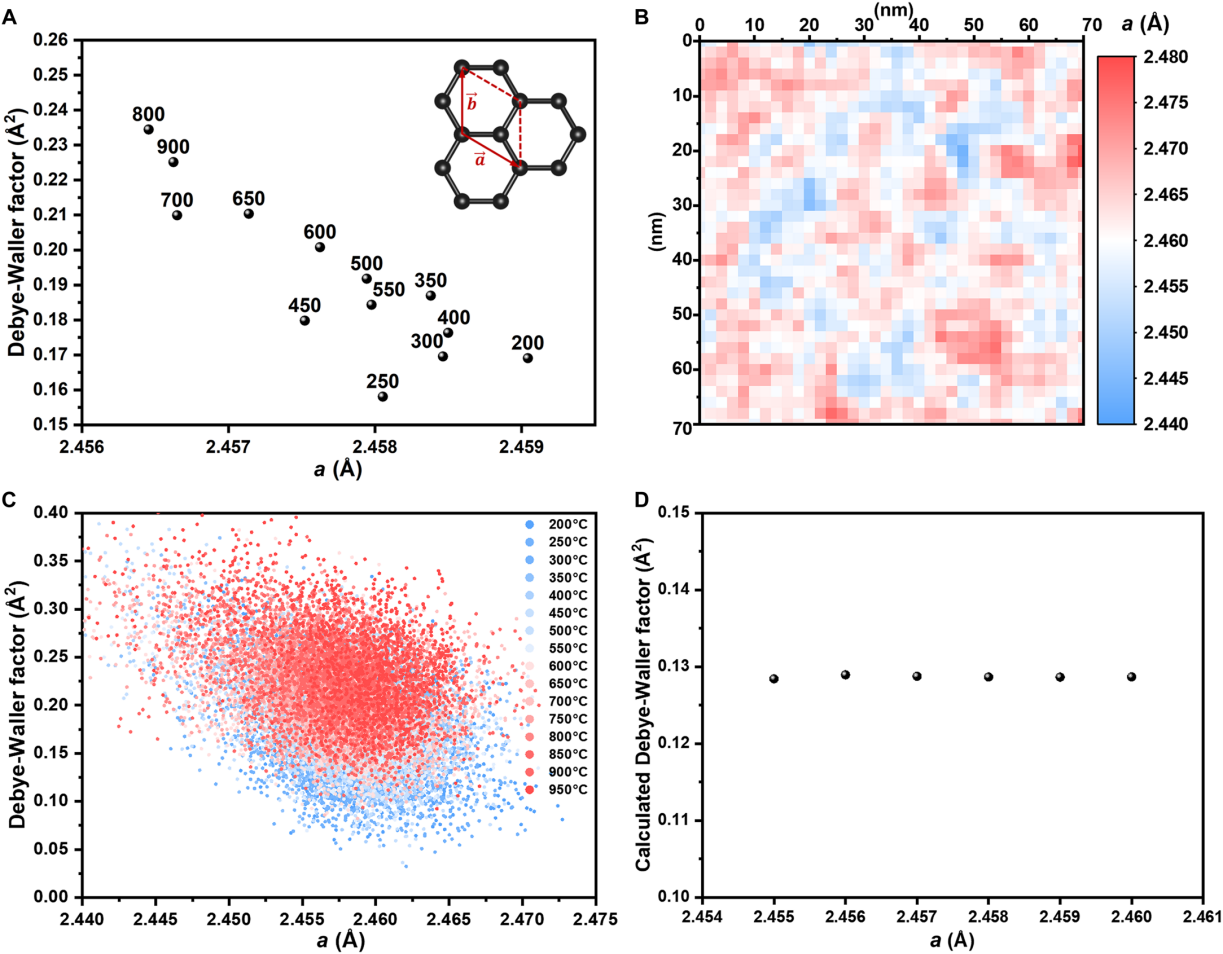
Analysis of the lattice parameter of monolayer graphene at different temperatures. (**A**) Debye-Waller factor of monolayer graphene as a function of its lattice parameter, *a* (3-by-3 binning). (**B**) Map of the lattice parameter mapping at 800°C. (**C**) Scatterplot between the Debye-Waller factor and the lattice parameter at all temperatures. (**D**) Debye-Waller factor as a function of monolayer graphene’s lattice parameter calculated by LAMMPS.

To study the thermal expansion of graphene and how the change in the lattice parameter could affect the Debye-Waller factor (*49*–*51*), we measure the lattice parameter from PED using AutoDisk (*29*). From 200° to 950°C, the lattice parameter of graphene changes from 2.459 to 2.456 Å, as shown in fig. S6. In Fig. 5A, the lattice parameter of monolayer graphene only changes by about 0.1%, while the Debye-Waller factor changes by about 33% from 0.169 to 0.225 Å^2^. Figure 5B shows the mapping of the lattice parameter at 800°C in the same region as in Fig. 4 (C and D); no spatial correlation is observed between the two maps. At each temperature from 200° to 950°C, the scatterplot of the Debye-Waller factor and lattice parameter is presented in Fig. 5C; again, both the lattice parameter and Debye-Waller factor change with temperature, but the two are not strongly correlated.

To better understand the impact of thermal expansion of graphene on the Debye-Waller factor, we calculate the phonon density of states corresponding to different atomic models (with 0.2% strain applied within the graphene plane) using LAMMPS (Large-Scale Atomic/Molecular Massively Parallel Simulator) (fig. S7). Figure 5D shows the variation of calculated Debye-Waller factor with the lattice parameter. The calculated Debye-Waller factor changes by about 0.4% when the lattice parameter changes from 2.455 to 2.460 Å.

Both experimental and theoretical results show that the lattice change of monolayer graphene has almost a negligible effect on the measurement of the Debye-Waller factor. The Debye-Waller factor changes at a faster rate of about 0.057%/°C, more than 400 times higher than the change of lattice parameter (0.00013%/°C). In addition, the lattice change is strongly constrained by the local atomic bonding, which may not be directly related to temperature. Given that vibrational characteristics directly reflect temperature, temperature measurement using the Debye-Waller factor with scanning PED is more sensitive, reliable, and capable of offering a nanometer-scale spatial resolution.

### Influence of graphene layer thickness on atomic vibrations

**Fig. 6.**
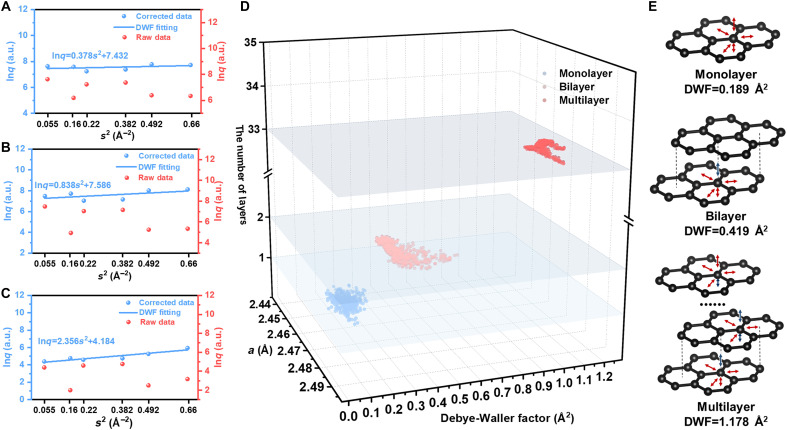
Debye-Waller factor of different-layer graphene at room temperature. Wilson plot (**A** to **C**) of the selected area (see figs. S8, areas 1 and 2, and S10) of monolayer, bilayer, and multilayer graphene. (**D**) Distribution of the measured Debye-Waller factor from monolayer, bilayer, and multilayer graphene. (**E**) Models of monolayer, bilayer, and multilayer graphene and arrows illustrating the vibration of atoms.

In addition to lattice thermal expansion, to investigate how the Debye-Waller factor changes with graphene thickness, we perform scanning PED on monolayer, bilayer, and multilayer graphene at room temperature (Fig. 6). An area with clean and uniform bilayer graphene was selected for analysis as shown in fig. S8. Like monolayer graphene, the local surface tilt in bilayer graphene also influences the intensity of diffraction spots. Using the same approach to quantifying the local surface curvature of monolayer graphene, we measured the surface morphology of bilayer graphene and calculated the Debye-Waller factor on the basis of correction factors considering the local tilt. The local tilt angle of the bilayer graphene and its analysis are shown in fig. S9.

A multilayer graphene film is prepared by mechanically exfoliating thick graphite flakes. To identify the number of layers, position-averaged convergent beam electron diffraction (*52*) is collected in experiment and compared with simulated patterns (fig. S10). The graphite is measured to be 11 ± 1 nm in thickness. For AB-stacking multilayer graphene, the *L* of an odd or even number of layers (*N*_layer_ = 33 ± 2 layers) is 15.95 or 16, respectively; we use 16 during the fitting (*53*). For all three samples, monolayer graphene, bilayer graphene, and thick graphite, as we expect, the Wilson plot fits well with a linear relationship after correction of the thickness-specific *L*, while the uncorrected ones do not in Fig. 6 (A to C). Debye-Waller factors are measured using correction factors *L* from Table 1, as shown in fig. S11. The measured Debye-Waller factors are 0.189, 0.419, and 1.178 Å^2^ for monolayer, bilayer, and multilayer graphene, respectively. The distribution of the measured Debye-Waller factor is shown in Fig. 6D. Note that the Debye-Waller factor of bilayer and multilayer graphene is 2.217 times and 6.233 times that of the monolayer graphene, respectively.

To understand the change in measured Debye-Waller factor in graphene with different thicknesses, we focus on the atomic bonding characteristics in layered materials. Depending on the vibration direction, the vibration modes can be divided into the planar (in-plane) and *z* (out-of-plane) modes. In monolayer graphene, the carbon atoms have bonding only within the atomic plane; vibration along the out-of-plane direction is much stronger. Theoretical calculations of the mean square atomic displacements are $u_{\mathrm{xy}}^{2}\left(0K\right)$ = 15.9 pm^2^ and $u_{z}^{2}\left(0K\right)$ = 40.4 pm^2^ (*54*). In bilayer graphene, this out-of-plane vibration is much compressed and transferred to the in-plane direction because of the coupling between the contacting surfaces. For a demonstration, in Fig. 6E, the loss of the bottom surface in the top layer, the loss of the top surface in the bottom layer, and the confinement applied against each surface reduce the *z*-mode vibration strength. The corresponding vibration energy can be transferred to the planar mode. The total vibrational energy stays the same as it is determined by the kinetic energy as a function of temperature. This effect is more pronounced in graphite with a higher thickness, as most of the *z*-mode vibration is suppressed with the loss of surface. With a higher thickness, the value of the Debye-Waller factor will quickly approach that of the bulk. Therefore, the Debye-Waller factor measurement of graphene is not only sensitive to temperature but also reflects the vibration characteristics.

## DISCUSSION

We have demonstrated temperature measurement with a nanometer-scale spatial resolution using scanning PED. By applying a corrected Wilson plot to the diffraction intensities, the Debye-Waller factor of graphene was determined and shown to be highly sensitive to temperature. With appropriate acquisition conditions and correction for surface tilt, the resolution of temperature mapping is ultimately limited only by the size of the electron probe—reaching the single-nanometer level, far surpassing conventional macroscopic techniques such as Raman spectroscopy or thermocouples. In terms of measurement precision, the Debye-Waller factor is also substantially more sensitive to temperature variations than thermal expansion, highlighting its potential as a more reliable nanoscale thermometer.

In particular, the observed dependence of the Debye-Waller factor on graphene layer thickness reveals the transfer of vibrational energy between out-of-plane (*z*) modes and in-plane (planar) modes, modulated by interlayer van der Waals interactions. This result underscores that Debye-Waller factor measurements not only quantify temperature but also provide insights into vibrational characteristics influenced by layer thickness, surface curvature, and other intrinsic material properties. Moreover, the ability to separate thermal effects from morphology-induced variations establishes a pathway to disentangle temperature-driven dynamics from structural distortions in two-dimensional systems.

Beyond graphene, this framework can be generalized to a wide class of materials. Two-dimensional crystals, van der Waals heterostructures, oxide interfaces, and even nanoscale electronic devices where thermal management is a critical bottleneck may all benefit from this approach. This diffraction-based temperature mapping method is best suited for single-crystalline, monoelement materials such as graphene and silicon, where corrected Wilson plots can be reliably constructed under quasikinematic conditions (fig. S12 and table S1). For multielement single crystals, the varying atomic form factors complicate the Wilson plot approach, and alternative techniques like quantitative convergent beam electron diffraction or direct Debye-Waller factor fitting using scanning PED are more appropriate (*55*, *56*). In polycrystalline samples, the presence of multiple zone axes precludes the definition of consistent correction factors, and in amorphous systems, the lack of discrete Bragg spots limits the applicability of diffraction-based analysis. Furthermore, the method is best applicable to sample thicknesses that preserve quasikinematic scattering, although thicker samples such as graphite of 10 nm in thickness can be addressed using PED to mitigate dynamical effects. An advantage of this approach is its compatibility with nanoscale scanning probes, enabling localized temperature measurements even across heterogeneous interfaces, where the spatially resolved Debye-Waller factor on each side of the interface can be analyzed separately.

By providing quantitative and spatially resolved thermal information, corrected PED-based Debye-Waller factor mapping opens opportunities for studying anharmonic effects, thermal transport, and energy dissipation at the near-atomic scale. More broadly, it establishes a robust and versatile strategy for quantitative thermal metrology in nanostructures, bridging the gap between fundamental lattice dynamics and device-level performance.

## MATERIALS AND METHODS

### Synthesis of graphene

Monolayer graphene was grown on Cu-Ni alloy foils containing 15 at % Ni (Cu_85_Ni_15_) through CVD. Before growth, the Cu-Ni foils were cleaned with hydrochloric acid, acetone, isopropanol, and deionized water to remove the surface oxide and organic impurity. The Cu-Ni substrate was then loaded into a quartz tube with a diameter of 100 mm. Cu in the foil could inhibit the formation of amorphous carbon on graphene, resulting in superclean surfaces (*27*). Before CVD, the substrates were annealed at 1050°C for 2 hours in a H_2_ and Ar flow [H_2_/Ar = 50/1000 standard cubic centimeter per minute (SCCM)] at atmosphere pressure to improve their surface flatness. The grains of the polycrystal Cu-Ni alloy also grew in size. During CVD, 10 to 20 SCCM methane (5% CH_4_ diluted in Ar), 30 SCCM H_2_, and 500 SCCM Ar were fed into the system. Monolayer graphene domains were obtained after exposure to methane for about 5 to 10 min. The system was cooled down naturally to room temperature in a mixed Ar/H_2_ flow. A Cu_85_Ni_15_ alloy foil was used as the substrate for the CVD growth of multilayer graphene (*28*).

### Characterization of graphene

Characterization of graphene was carried out using TEM (Thermo Fisher Scientific Spectra 300 aberration-corrected STEM operated at 80 kV; see details in fig. S13) and Raman spectroscopy (Horiba LabRAM Solei, 532 nm; see details in fig. S14).

### Sample preparation for in situ scanning PED

Graphene grown on Cu or Cu_85_Ni_15_ foils obtained according to the above operations was transferred to the in situ heating chips (E-FHDC-VO, Protochips), SiO_2_/Si substrates, or TEM grid by a wet transfer method. The graphene film was spin-coated with monomethylamine (MMA) and poly(methyl methacrylate) (PMMA) (2000r) and then baked at 120°C for 2 min. After that, a supersaturated solution of FeCl_3_ was used to remove the Cu or Cu_85_Ni_15_ foils. After being washed with deionized water, PMMA/MMA/graphene was subsequently placed onto the substrates. Last, PMMA and MMA were dissolved with acetone.

For a thicker graphite sample, the graphite flakes obtained by mechanically stripping the oriented graphite with a tape were first attached to the Si wafer spin-coated with PMMA and held at 120°C for 2 min. Then, the Cu grid was placed on the graphite sheet under an optical microscope and held at 150°C for 5 min, followed by the removal of PMMA using acetone.

### Scanning PED

PED was performed using a double aberration-corrected Thermo Fisher Scientific Spectra 300 (S)TEM operated at 300 kV and a TopSpin system from NanoMEGAS. The diffraction patterns were recorded using a 256 by 256–pixel Quantum MerlinEM detector. During the temperature mapping experiments, each diffraction pattern was acquired with a 10-ms exposure time. To enhance the signal-to-noise ratio, we applied spatial binning (e.g., 15 by 15), resulting in an effective total exposure time equivalent to 2.25 s per binned point. Different precession angles (0.02° for graphene and 2° for thicker graphite) were used. In situ heating experiments were performed using Fusion Select Systems from Protochips. To analyze the experimental scanning PED data, we developed a Python script on the basis of the modified version of AutoDisk originally described in (*29*). The updated AutoDisk package and manual are now available at https://github.com/yk0109/DWF-ED_analysis.git.
