# Supplementary material for: Nanoscale temperature mapping through thermal vibration characterization using scanning precession electron diffraction
Source: Sci Adv. 2026 Feb 13;12(7):eaeb9234. doi: 10.1126/sciadv.aeb9234 (PMC12904201; doi:10.1126/sciadv.aeb9234)
Supplement: Supplementary file 1 — Supplementary Text Figs. S1 to S14 Table S1 [file sciadv.aeb9234_sm.pdf]

Science Advances
AAAS

Supplementary Materials for

Nanoscale temperature mapping through thermal vibration characterization using scanning precession electron diffraction

Kun Yang *et al.*

Corresponding author: Wenpei Gao, gaowenpei@sjtu.edu.cn; Tao Deng, dengtao@sjtu.edu.cn; Hui Wang, huiwang@csu.edu.cn; Tianru Wu, trwu@sjtu.edu.cn

Sci. Adv. **12**, eaeb9234 (2026)
DOI: 10.1126/sciadv.aeb9234

This PDF file includes:

Supplementary Text
Figs. S1 to S14
Table S1

Supplementary Materials

Derivation of the Wilson Plot

The structure factor can be described as,

$$F(hkl) = \sum_{j=1}^{n} f_j(s)\, e^{2\pi i(hx_j+ky_j+lz_j)} e^{-Bs^2}, \tag{S1}$$

in which $f_j(s)$ is the atomic form factor for atom j, given by Weickenmeier(*25*), (x_j, y_j, z_j) is the position of atom j, (h, k, l) is the Miller indices of the g_{th} order diffraction disk, e^{-Bs^2} is the usual Debye-Waller factor and sometimes B is simply called the Debye-Waller factor and s=g/2.

Simulation of the reciprocal rods

The reciprocal rods are derived from the three-dimensional Fourier transform of the electronic structure of monolayer graphene, calculated by VASP. The radius of Ewald sphere is based on the electron acceleration voltage (300kV). We use the atomic scattering factor to describe the attenuation of the Bragg peak for monolayer graphene in the direction of a parallel incident electron beam. Due to the curvature of the Ewald sphere, the areas where it intersects the reciprocal rods are different.

Molecular Dynamics Calculations

Large-scale Atomic/Molecular Massively Parallel Simulator (LAMMPS) was used to calculate the planar thermal mean square displacement of an atom in graphene, $\langle u_p^2 \rangle$. The Debye-Waller factor can be expressed as,

$$\mathrm{B} = 8\pi^2 \langle u_p^2 \rangle, \tag{S2}$$

where $\langle \ldots \rangle$ denotes the thermal average. In the harmonic approximation, it is given by (*29*),

$$\langle u_p^2 \rangle = \frac{\hbar}{2m} \int_0^{\omega_m} coth \frac{\hbar\omega}{2k_B T} \cdot \frac{g_p(\omega)}{\omega} d\omega \,, \tag{S3}$$

in which m is the mass of a carbon atom, $g_p(\omega)$ is the normalized planer phonon density of states (PDOS) and ω_m is the maximum phonon frequency.

$$\langle u_p^2 \rangle = \langle u_{xy}^2 \rangle cos^2\theta + \langle u_z^2 \rangle sin^2\theta \tag{S4}$$

in which θ is tilt angle of monolayer graphene.

Measuring Local Tilt of Graphene

For a flat graphene sample perpendicular to the incident electron beam, the intensity of the diffraction disks of the same order in the diffraction pattern should be the same. To probe local tilt variations, we focused on second-order diffraction spots of graphene, which offer improved signal-to-noise ratio (as shown in Fig. S1). Graphene's diffraction pattern exhibits 6-fold rotational symmetry. As illustrated in Fig. S3B, we assigned six rotational axes (ω_1 to ω_6) and categorized them into two groups. We further grouped the six diffraction spots into three pairs and compared their relative intensities (see Fig. S3A). The calculations revealed that diffraction spots located further from the tilt axis exhibit greater

intensity variation than those closer to the axis. To determine the tilt axis, again, we divided these six diffraction spots into three pairs and compared their intensity relationships.

For example:

- If $I_{R_0+R_3} \approx I_{R_2+R_5} \gg I_{R_1+R_4}$, the tilt axis is ω_1;
- If $I_{R_0+R_3} \gg I_{R_2+R_5} \approx I_{R_1+R_4}$, the tilt axis is ω_2.

Based on this evaluation, we obtain the tilt axes in the local region, as shown in Fig. S3C and S9C. Furthermore, to quantify the tilt angle, we calculate $\frac{2(R_1+R_4)}{R_0+R_2+R_3+R_5}$ for $\omega_{1,3,5}$ and $\frac{R_1+R_2+R_4+R_5}{2(R_0+R_3)}$ for $\omega_{2,4,6}$. Applying these calculations, we generate a quantitative tilt map, as presented in Fig. S3D.

For the AB-stacking bilayer graphene structure model, we calculated the shape of the same-order Bragg peak in the k_Z-direction, as shown in Fig. S9B. Subsequently, tilt the sample, which is equivalent to tilting the Ewald sphere, and calculate the relationship between the tilt angle and the diffraction intensity ratio. Based on the intersection position of the Ewald sphere and the reciprocal rod, we could obtain the corresponding calibration curve, as shown in Fig. S9A. For the bilayer region selected in Fig S8A (area 2), excluding the region covered by amorphous contaminants, the angle distribution of this region can be measured using the calibration curve.

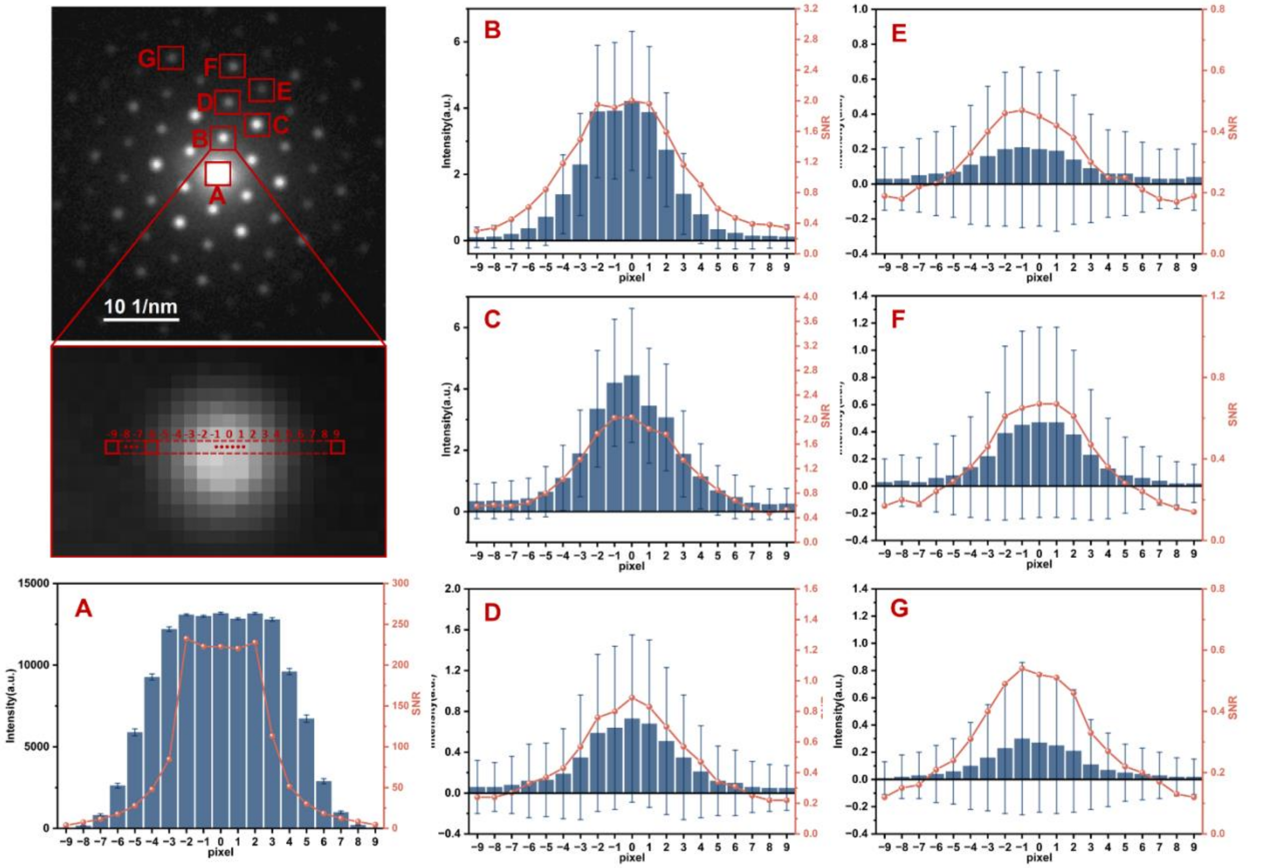

Fig. S1. Quantifying diffraction intensity from experimental diffraction patterns. A-E are the means and standard deviations of which are counted respectively and thus the signal-to-noise ratio (SNR) is calculated.

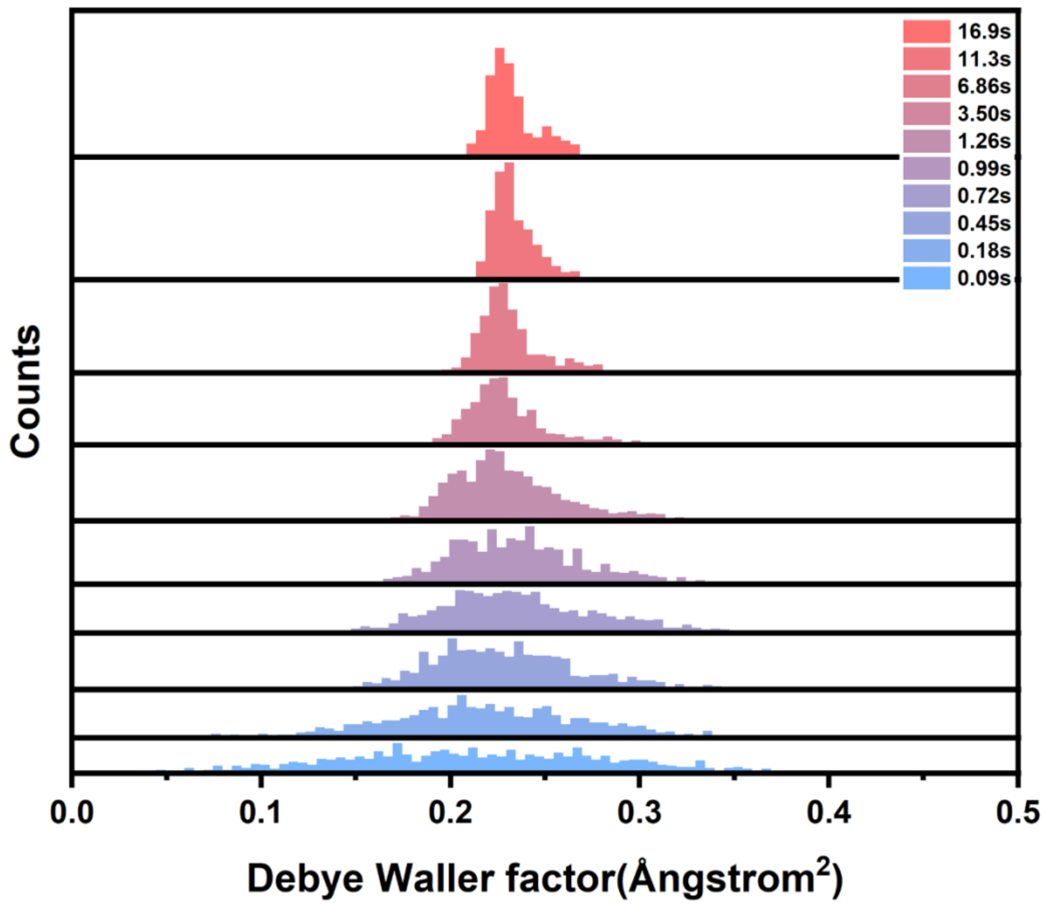

Fig. S2. The histogram of Debye-Waller factor of different exposure time at 800 °C.

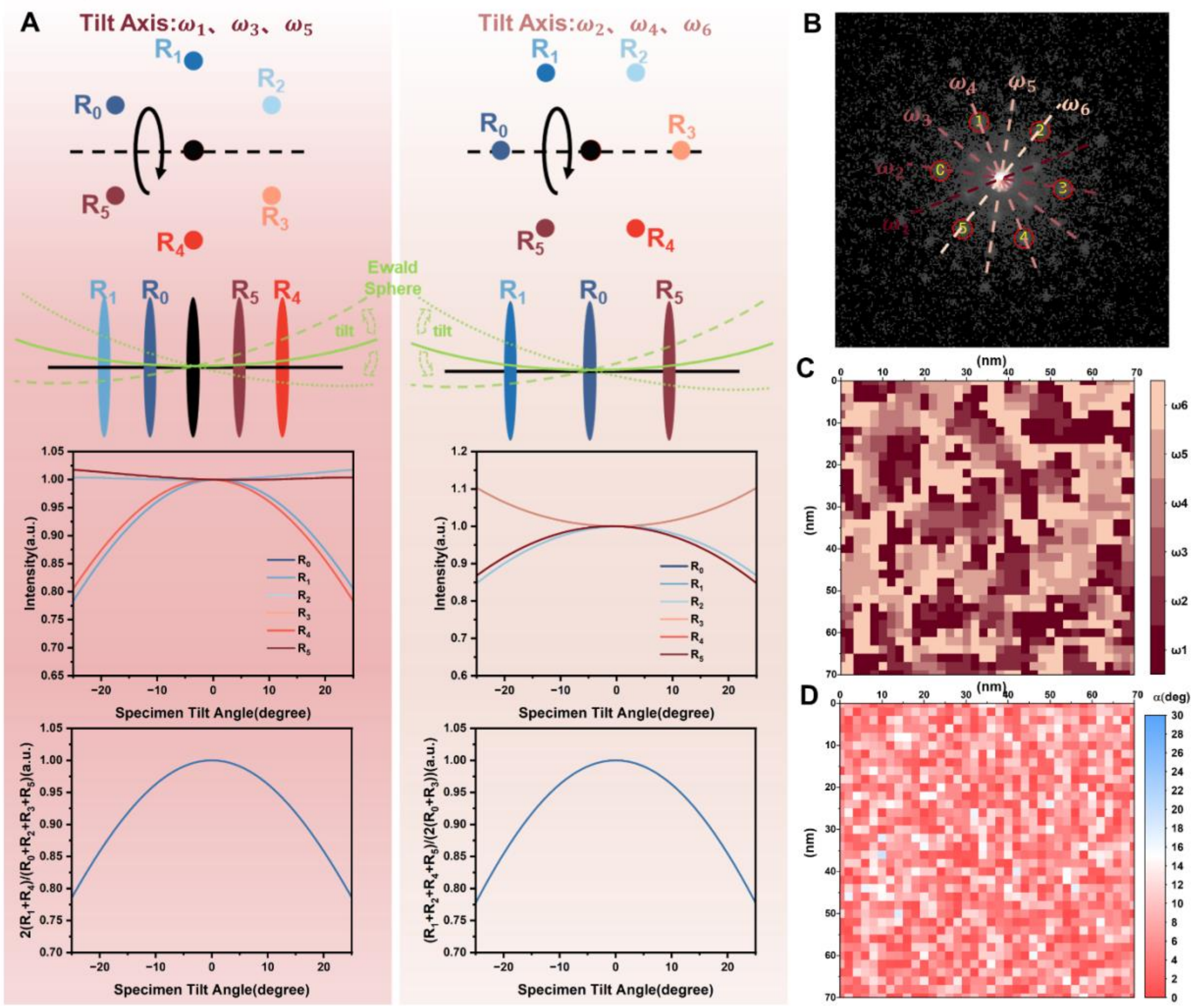

Fig. S3. Schematic diagram for analyzing the tilt angle of monolayer-graphene. (**A**) The schematic diagram of the reciprocal rods and the tilt axes and the diffraction intensity ratio as a function of tilt angle. (**B**) The diffraction pattern of monolayer graphene and the rotational axes (ω_1~ω_6). (**C**) The cluster map of the rotational axes. (**D**) The tilt angle map of the select area at 800 °C.

Note that, for the ω_1, ω_3, ω_5 tilt axis, we calculate the $\frac{2(R_1+R_4)}{R_0+R_2+R_3+R_5}$ value of the simulations and experiments, while for ω_2, ω_4, ω_6 tilt axis, we use the $\frac{R_1+R_2+R_4+R_5}{2(R_0+R_3)}$ value.

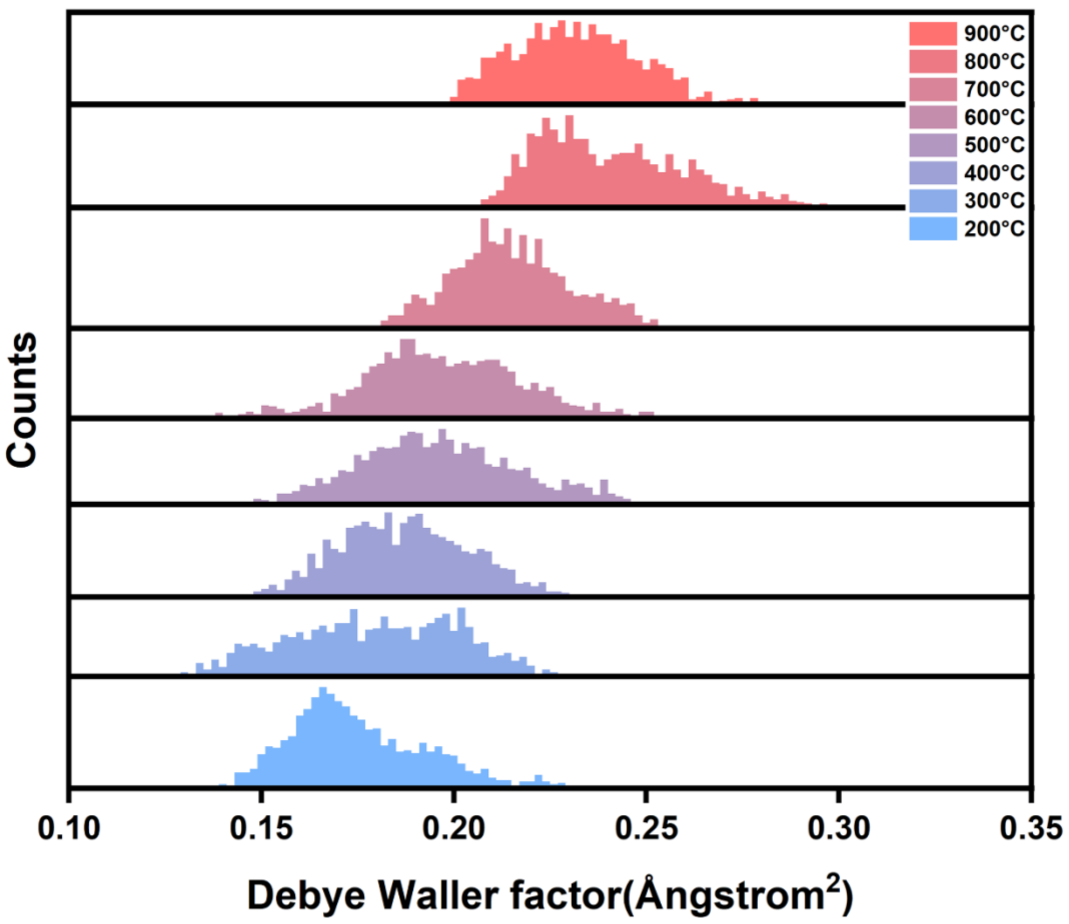

Fig. S4. The histogram of Debye-Waller factor of different temperature.

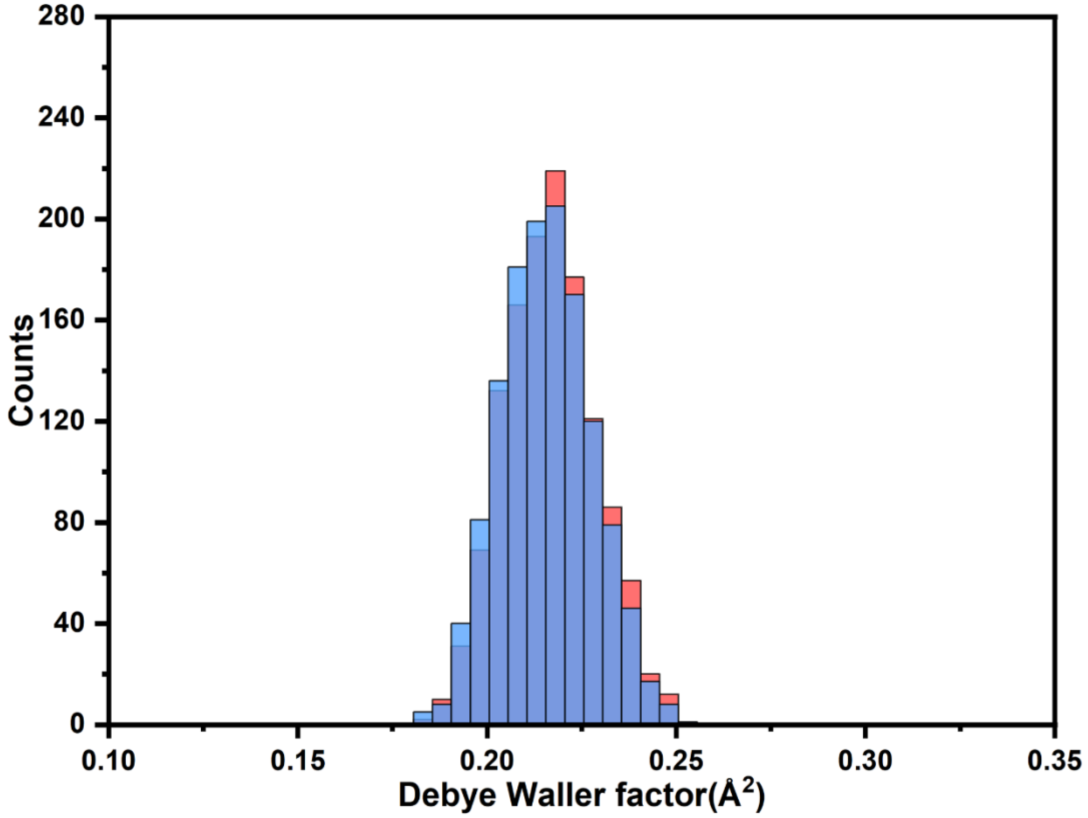

Fig. S5. The histogram of Debye-Waller factor at 800 °C before (red) and after (blue) correction.

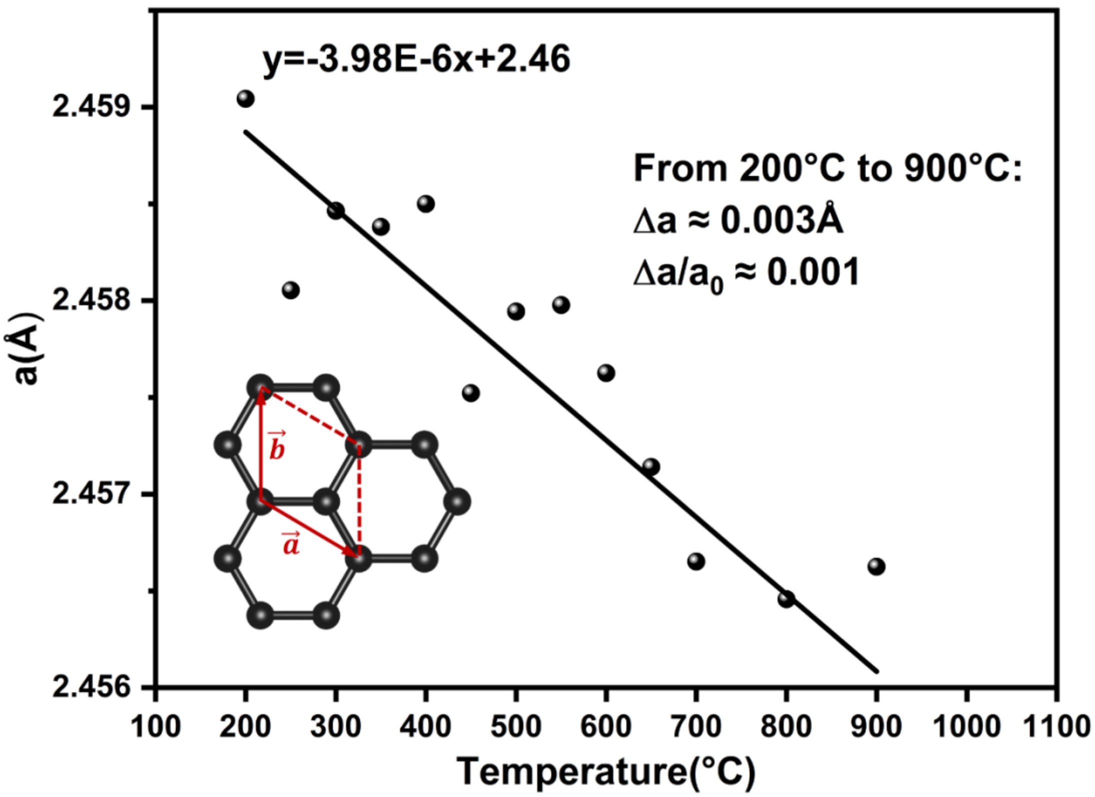

Fig. S6. The lattice parameter as a function of temperature.

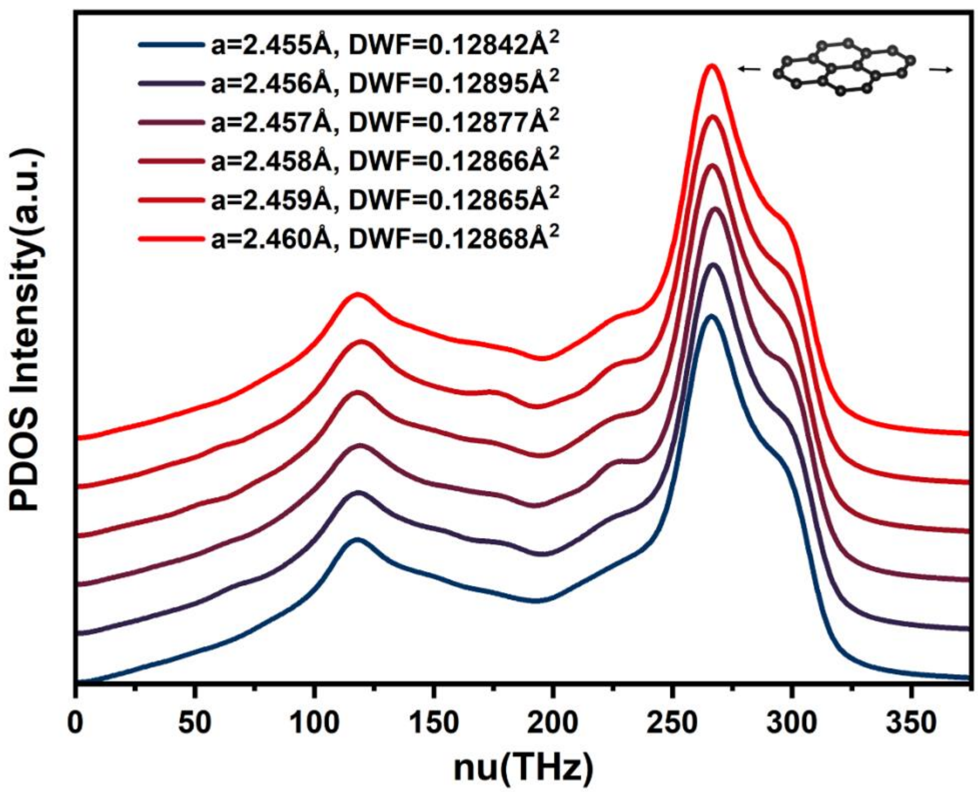

Fig. S7. The normalized planer phonon density of states of different lattice parameters.

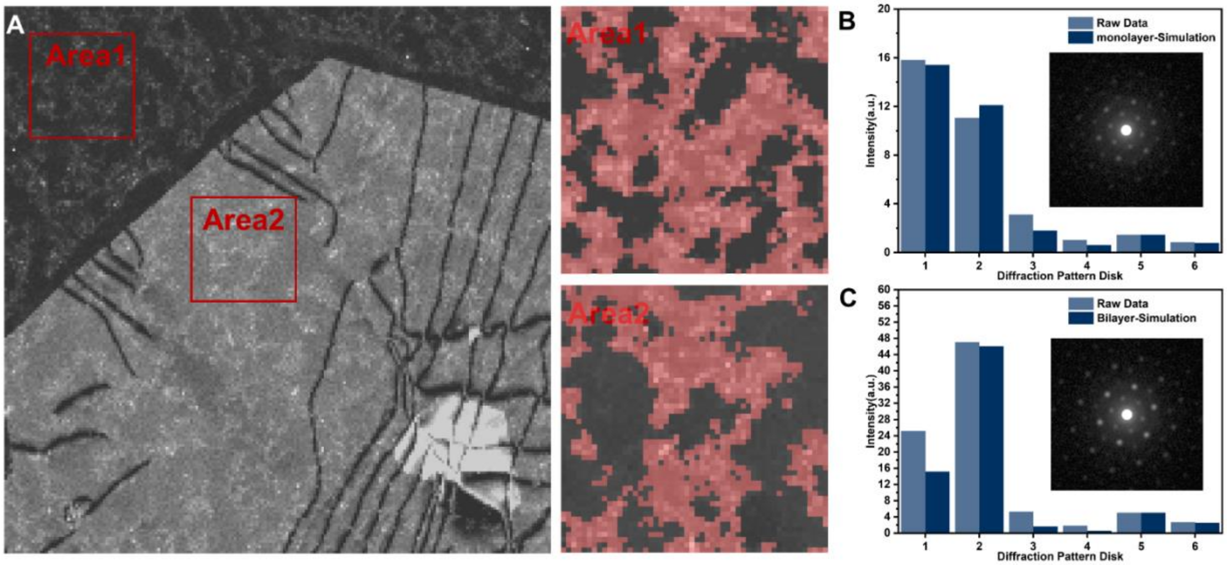

Fig. S8. Analysis of monolayer and bilayer graphene. (**A**)The ADF image of monolayer (area1) and bilayer (area2) graphene. Approximate areas of amorphous contaminants are marked in pink. (**B-C**) The diffraction intensity of experimental and simulation data and the diffraction patterns of area1 and area2.

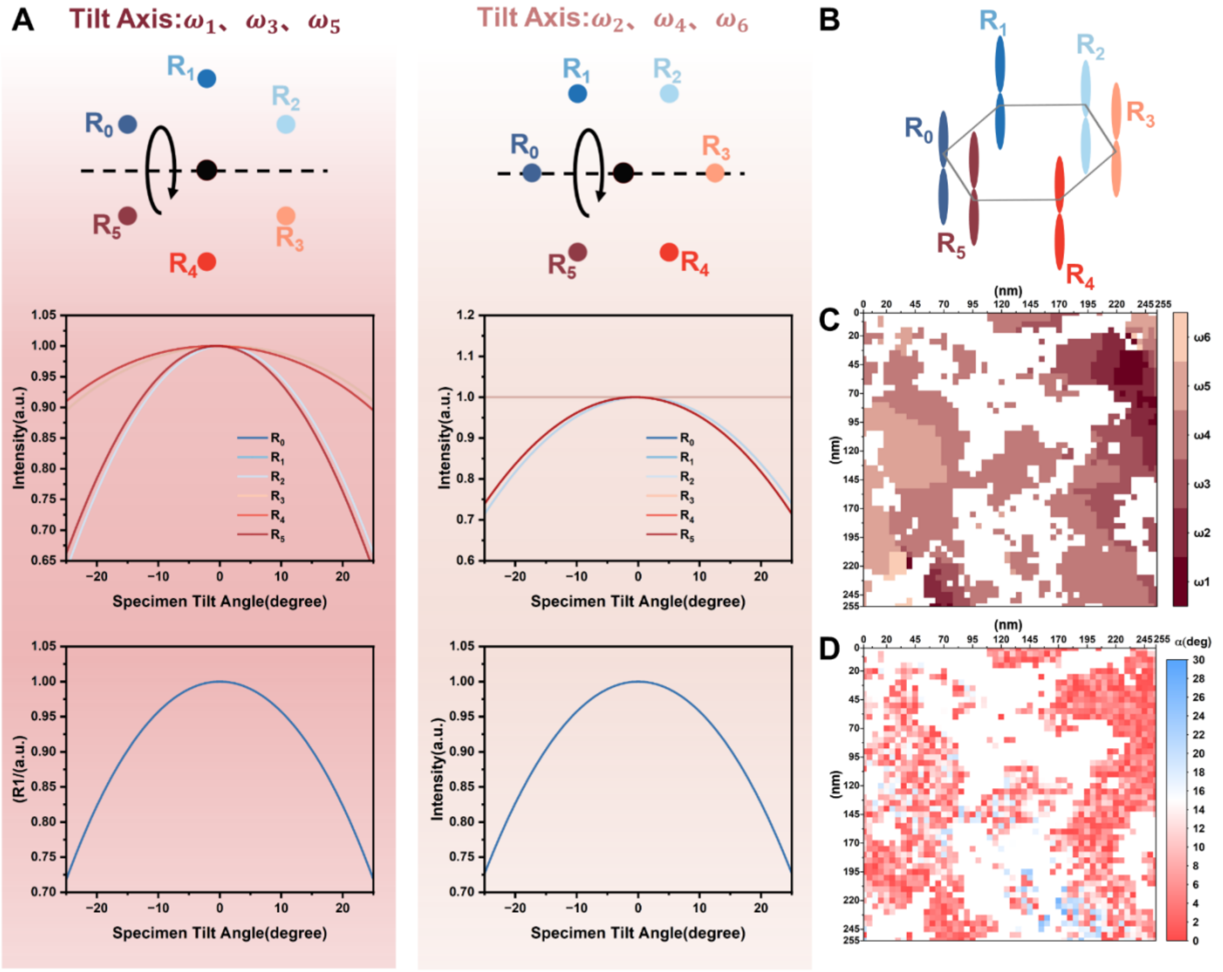

Fig. S9. Schematic diagram for analyzing the tilt angle of bilayer-graphene. (**A**) The schematic diagram of the reciprocal rods and the tilt axes and the diffraction intensity ratio as a function of tilt angle (**B**) The intensity distribution of same-order diffraction spots of bilayer graphene along k_Z-direction. (**C**) The cluster map of the rotational axes. (**D**) The tilt angle map of the select area at room temperature.

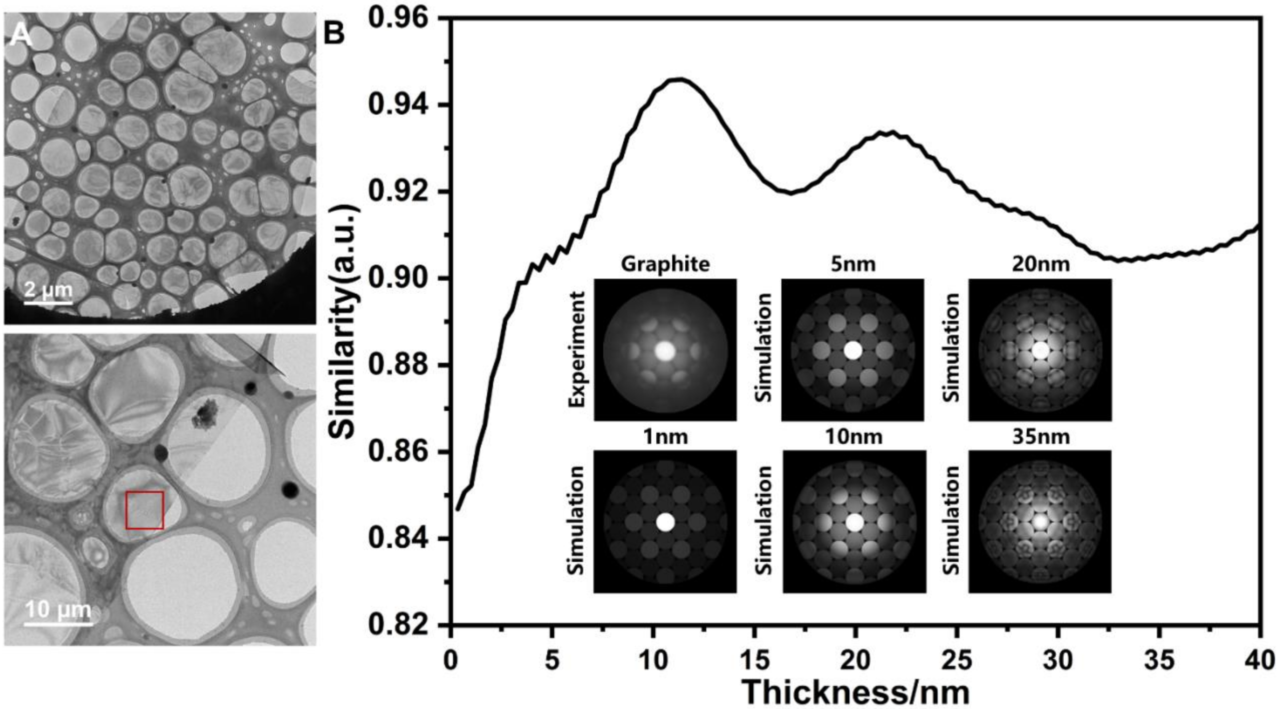

Fig. S10. Measuring multilayer-graphene thickness using PACBED. (**A**) TEM image of multilayer-graphene. (**B**) the fitting of the experimental PACBED results with the simulated PACBEDs. The inset shows the PACBED acquired from the boxed region in the TEM image, and simulated PACBEDs of graphene with thicknesses from 0.4 nm to 40 nm.

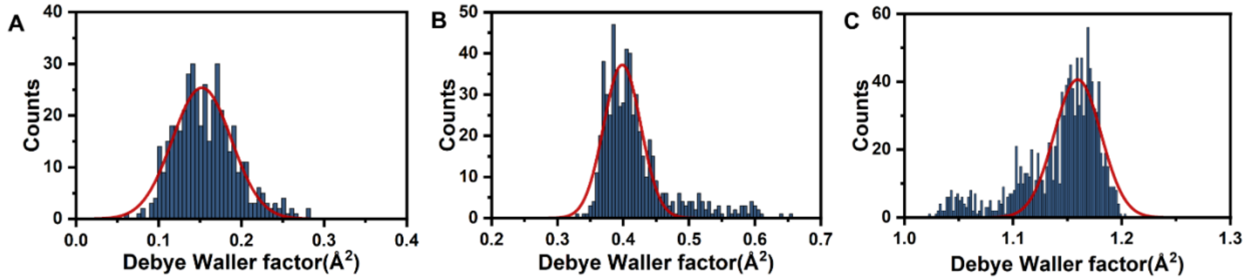

Fig. S11. The histogram of Debye-Waller factor. The histogram of Debye-Waller factor from (**A**) Monolayer graphene, (**B**) Bilayer graphene, and (**C**) Graphite.

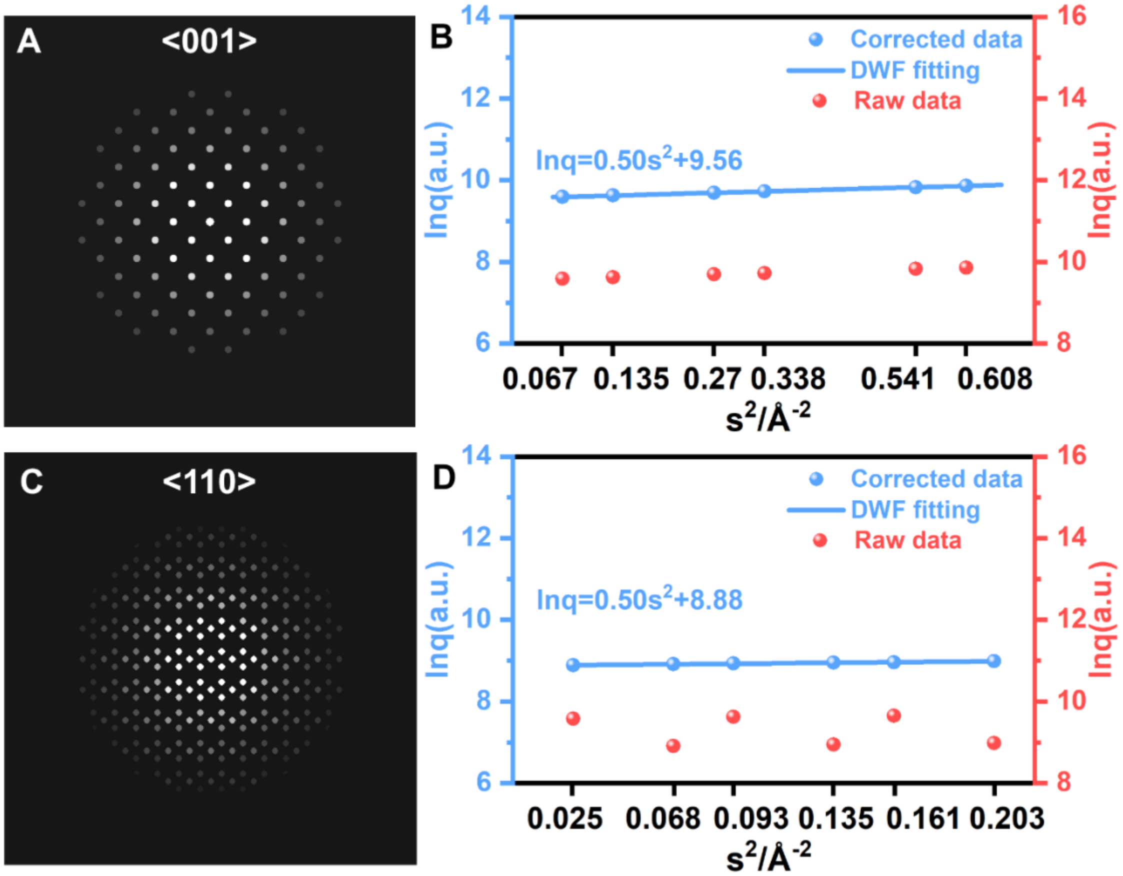

Fig. S12. The simulated diffraction patterns and the Wilson plots of silicon. (A) The <001> zone diffraction pattern of silicon. **(B)** The Wilson plot of silicon<001> using the correction factor, L, from Table S1 and the results of linear fitting. (**C**) The <110> zone diffraction pattern of silicon. (**D**) The Wilson plot of silicon<110> using the correction factor, L, from Table S1 and the results of linear fitting.

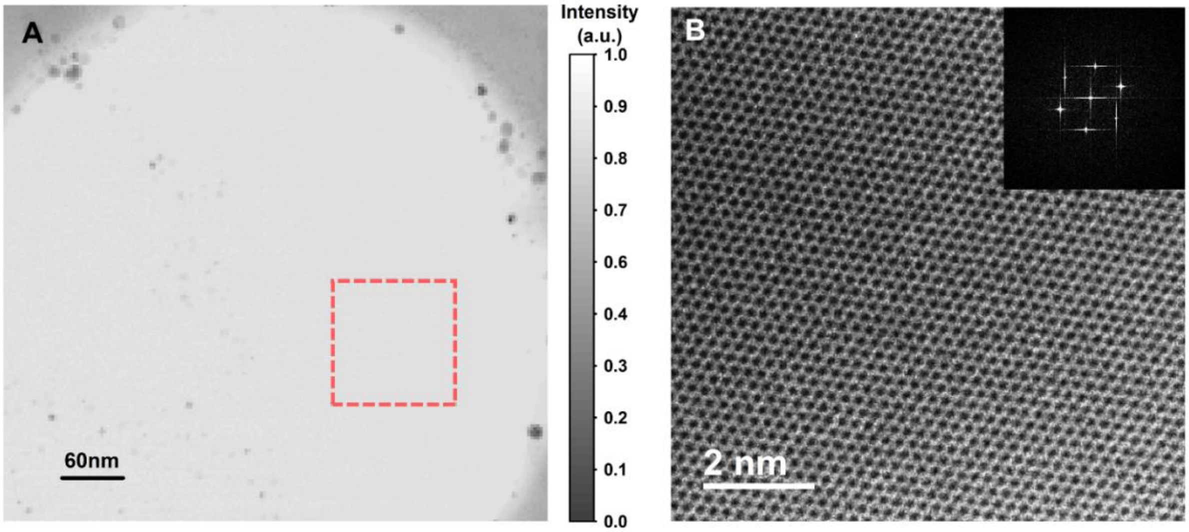

Fig. S13. Images of graphene. (**A**) The bright field (BF) image from 4D electron diffraction data by integrating the intensity of the annular region of 0-10 mrad from the diffraction pattern Fig. 1B. (**B**) The high-resolution electron microscopy (HREM) image of monolayer-graphene at 80kV.

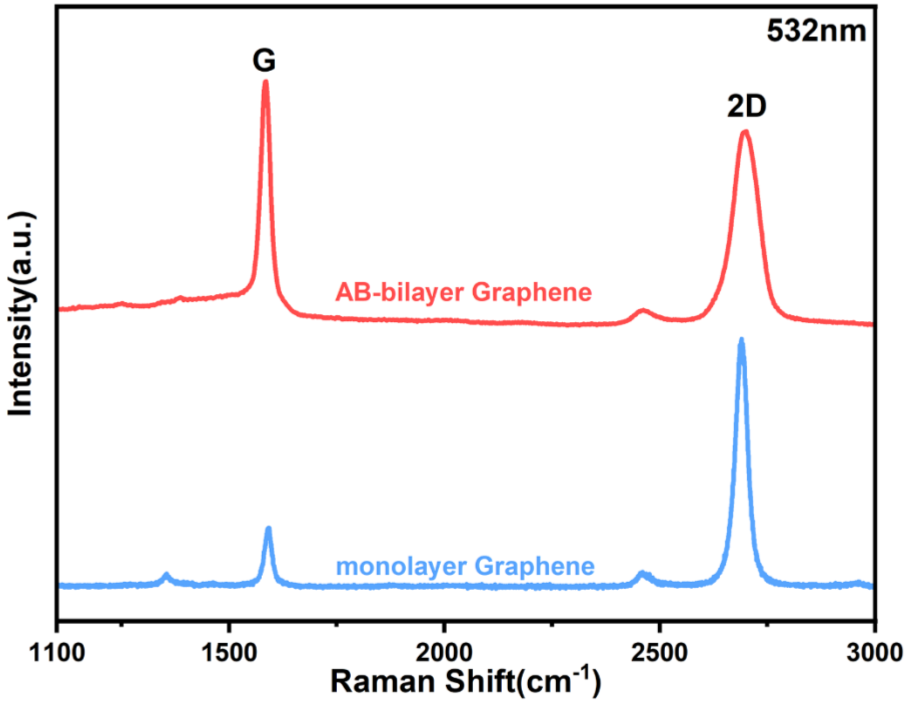

Fig. S14. Raman spectra of monolayer and bilayer graphene in the G and 2D mode regions under excitation at 532 nm.

Table S1. L of the g_{th} diffraction for silicon

crystal	1st	2nd	3rd	4th	5th	6th
Silicon<001>	1	1	1	1	1	1
Silicon<110>	1	2	1	2	1	2
